# TP53 modulates radiotherapy fraction size sensitivity in normal and malignant cells

**DOI:** 10.1038/s41598-021-86681-6

**Published:** 2021-03-29

**Authors:** Selvakumar Anbalagan, Cecilia Ström, Jessica A. Downs, Penny A. Jeggo, David McBay, Anna Wilkins, Kai Rothkamm, Kevin J. Harrington, John R. Yarnold, Navita Somaiah

**Affiliations:** 1 The Institute of Cancer Research, London, UK; 2 The Royal Marsden NHS Foundation Trust, London, UK; 3 Genome Damage and Stability Centre, University of Sussex, Sussex, UK; 4 University Medical Centre Hamburg-Eppendorf, Hamburg, Germany; 5 The Royal Marsden, Downs Road, Sutton, SM2 5PT UK

**Keywords:** Radiotherapy, DNA damage and repair

## Abstract

Recent clinical trials in breast and prostate cancer have established that fewer, larger daily doses (fractions) of radiotherapy are safe and effective, but these do not represent personalised dosing on a patient-by-patient basis. Understanding cell and molecular mechanisms determining fraction size sensitivity is essential to fully exploit this therapeutic variable for patient benefit. The hypothesis under test in this study is that fraction size sensitivity is dependent on the presence of wild-type (WT) p53 and intact non-homologous end-joining (NHEJ). Using single or split-doses of radiation in a range of normal and malignant cells, split-dose recovery was determined using colony-survival assays. Both normal and tumour cells with WT p53 demonstrated significant split-dose recovery, whereas Li-Fraumeni fibroblasts and tumour cells with defective G1/S checkpoint had a large S/G2 component and lost the sparing effect of smaller fractions. There was lack of split-dose recovery in NHEJ-deficient cells and DNA-PKcs inhibitor increased sensitivity to split-doses in glioma cells. Furthermore, siRNA knockdown of p53 in fibroblasts reduced split-dose recovery. In summary, cells defective in p53 are less sensitive to radiotherapy fraction size and lack of split-dose recovery in DNA ligase IV and DNA-PKcs mutant cells suggests the dependence of fraction size sensitivity on intact NHEJ.

## Introduction

For almost 100 years, radiation therapists have aimed to deliver the highest total dose to cancers using multiple small dose increments (1.8–2.0 Gy fractions) based on early observations that small fractions spare surrounding healthy tissues relative to cancers, typically squamous cell carcinomas^1,2^. Recent randomised clinical trials confirm that breast cancer is unusual in being more sensitive to fraction size than historically assumed, having an average sensitivity comparable to that of the surrounding healthy tissues of the breast, pectoral muscles and rib-cage^3–5^. The implications are that continued use of 1.8–2.0 Gy fractions spares breast cancer as much as the healthy tissues, undermining their long-assumed advantages. Standard treatment schedules for women with breast cancer increasingly use fewer, larger fractions delivered to a lower total doses over shorter overall treatment times, and it appears that prostate cancer also resembles breast cancer in this respect^6–8^. It is likely that primary cancers arising at other anatomical sites would also benefit from this approach, and recent evidence for an impact of fraction size on immune activation further heightens the importance of understanding this fundamental treatment parameter at cell and molecular levels^9–12^.

Clinical fractionation responses to ionising radiation are considered to have a cellular basis, tested by comparing clonogenic survival after single and split doses or high and low dose rate exposures^13^. Clonogenic survival increases when a single dose is split in two smaller fractional doses delivered several hours apart, and the degree to which this cellular recovery occurs is a measure of sensitivity to fraction size. At a subcellular level, the processing and repair of DNA double-strand breaks (DSB) are considered critical determinants of cell fate in normal and malignant cells^14^. Cell fate measured by clonogenicity correlates with unrejoined DSB and unbalanced chromosome exchanges between spatially and temporally related lesions, the latter probability increasing as a quadratic function of single dose or fraction size^15,16^. We postulate that the low fidelity DSB repair characteristic of non-homologous end-joining (NHEJ) explains the increased sensitivity to fraction size in dose-limiting late-responding normal tissues. These tissues including brain, lung, heart, liver, kidney and musculoskeletal tissues are characterised by very low proliferative indices with cells predominantly in G0 phase of the cell cycle, for which NHEJ is the dominant, if not exclusive, DSB repair system in humans^17–19^. This is also likely to be relevant in cancers such as breast and prostate with low proliferative indices^20,21^. Against this background, we explore the cellular basis of fractionation sensitivity in a range of normal human wild-type (WT)/mutant and malignant cell lines to test the hypothesis that fraction size sensitivity is modulated by TP53 and depends on error-prone NHEJ of radiation-induced DNA DSBs in G0/1 phase of the cell cycle.

## Materials and methods

### Establishment of primary fibroblasts

Breast primary skin fibroblast cultures were established and maintained as previously described^22^. Under an ethically approved protocol at the Institute of Cancer Research (IRAS project ID 163422, NRES Committee London—Queen Square: REC reference 14/LO/2301, Protocol number CCR4234), informed consent was obtained from all patients (patients < / = 18 years were excluded) to obtain skin samples from tissue removed at the time of routine surgery. Skin biopsies were collected in L-15 medium from a breast reduction mammoplasty specimen. Initially, tissue explants were harvested from the skin biopsy by removing excess fat and then sliced into 2 × 2 mm sections under sterile conditions. These tissue explants were transferred to a T25 flask and kept in a humidified incubator at 37 °C with 5% CO_2_. The breast skin fibroblast outgrowths were cultured in DMEM medium supplemented with 10% FBS (GIBCO), 50 μg/mL gentamicin (Life Technologies) and 2.5 μg/mL amphotericin B (GIBCO).

### Cell lines and irradiation treatment

Normal human cell lines used in this study include S009 breast primary skin fibroblasts (established in this lab as above); skin fibroblasts 1BR hTERT (WT), 411BR hTERT (DNA ligase IV-deficient) were obtained from Prof Jeggo (University of Sussex), and transformed Li-Fraumeni skin fibroblasts MDAH041 (p53 184FS) obtained from Prof Tainsky (Karamanos Cancer Institute). Tumour cell lines, including M059K (p53-mutant, WT DNA-PKcs), M059J (p53-mutant, DNA-PKcs defective) glioma cells, LNCaP (WT p53), PC3 (p53-mutant) prostate and A2780 WT and A2780/E6 (HPV E6-induced silencing of p53) ovarian cells, were obtained from Prof Harrington (The Institute of Cancer Research). Cells were cultured in DMEM medium except LNCaP, PC3 and A2780 (RPMI-1640) supplemented with 10–15% FBS (GIBCO) in a humidified incubator at 37 °C with 5% CO_2_. All cell lines were routinely mycoplasma tested with MycoAlert Mycoplasma detection kit (Lonza). For irradiation experiments, cells were exposed to either acute single doses or fractionated radiation (split-dose 8 h apart or daily fractions) at room temperature using 250 kV X-rays at a dose-rate of 0.587 Gy/min (AGO, Reading, UK).

### Colony survival assay

Primary fibroblasts were plated onto feeder cells and allowed to adhere for 24 h prior to irradiation with either single acute doses or equally-split doses 8 h apart and allowed to form colonies as previously described^23^. The tumour cell lines were plated as indicated before irradiation (either acute single doses or split-dose 8 h apart or once-daily fractions as indicated) and colonies counted after 10–21 days by staining with 1% methylene blue (in 70% methanol). Colonies having more than 50 cells were counted and plating efficiency determined. Surviving fractions were calculated and data plotted using GraphPad Prism v7.0d (GraphPad Software Inc., La Jolla, CA) as previously described^24^. Recovery factor (RF) defined as the ratio of split-dose to single-dose survival was calculated as a measure of sensitivity to fraction size^13^. All colony survival assays were carried out a minimum of 3 repeats. Error bars represent standard errors from at least 3 independent experiments. Significance: ‘*’ p-Value < 0.05; ‘ns’ non-significant.

### Cell cycle analysis

Either 0.3 × 10^5^ cells or 2 × 10^5^ cells were seeded and irradiated the following day as indicated. Cells were harvested by trypsinisation, fixed in ice-cold ethanol and stained with propidium iodide (PI) solution (PI 10 μg/mL, RNAse 100 μg/mL; Sigma Aldrich). FACS analysis was performed for 10,000 cells per experimental condition using BD LSR II flow cytometer (BD Bioscience) and the data were analysed using BD FACSDiva v8.0.1 (BD Bioscience).

### Western blotting (WB)

Protein lysates were extracted using UTB (9 M urea, 0.75 M Tris–HCL [pH 7.5] and 0.15 M β-mercaptoethanol) and WB was performed as previously described^24^. Primary antibodies used were p53 DO-7 (DAKO, #M7001), phospho-p53 (ser15) (Cell signalling, #9284), phospho-H2AX (ser139) (Millipore, #05-636, clone JBW301), H2AX (Millipore, AB10022), p21 (Cell signalling, #2947), GAPDH (Novus Bio, #NB600-502). Secondary antibodies used were Alexa-Fluor 680 goat anti-mouse IgG (Invitrogen), Alexa-Fluor 680 goat anti-rabbit IgG (Invitrogen), IRDye 800CW donkey anti-mouse IgG (LI-COR) and IRDye 800CW donkey anti-rabbit IgG (LI-COR). All membranes were scanned using the Odyssey Imager and images acquired using Odyssey software (LI-COR Biosciences, Lincoln NE).

### Gene knockdown assay

All siRNA transfections were performed using Dharmacon transfection protocol according to manufacturer’s instructions. Briefly, 2 × 10^5^ cells were seeded and allowed to adhere for 24 h. Plated cells were transfected with 15 nM siRNA using DharmaFECT1 transfection reagent. Transfection medium was replaced with complete medium 24 h and 48 h post-transfection; transfected cells were used for colony survival assay as indicated. siRNA for p53 was obtained from Dharmacon (ON-TARGETplus #L-003329-00-0005) and negative control from Dharmacon (ON-TARGETplus Non-targeting Control Pool, #D-001810-10-05).

### Drug treatments

The DNA-PKcs inhibitor KU0064648 (obtained from Prof Harrington, The Institute of Cancer Research) was used at the indicated concentrations and radiation doses. For colony survival assays involving KU0064648, plated cells were treated with either DMSO or KU0064648, 1 h before exposure to acute or split-dose radiation and were allowed to form colonies without replacing the media.

### Statistics

Statistical significance was calculated using one-tailed student’s t-test and a p-value ‘*’ < 0.05 was considered to be statistically significant and ‘ns’ non-significant. Error bars indicate the SEM and ‘±’ the SD of three individual experiments performed in triplicate. All methods and experimental protocols described above were carried out in full accordance with the guidelines and standard operating procedures at The Institute of Cancer Research and The Royal Marsden NHS Foundation Trust.

## Results

### p53-competent primary fibroblasts show fraction size sensitivity as evidenced by split-dose recovery

**Figure 1:**
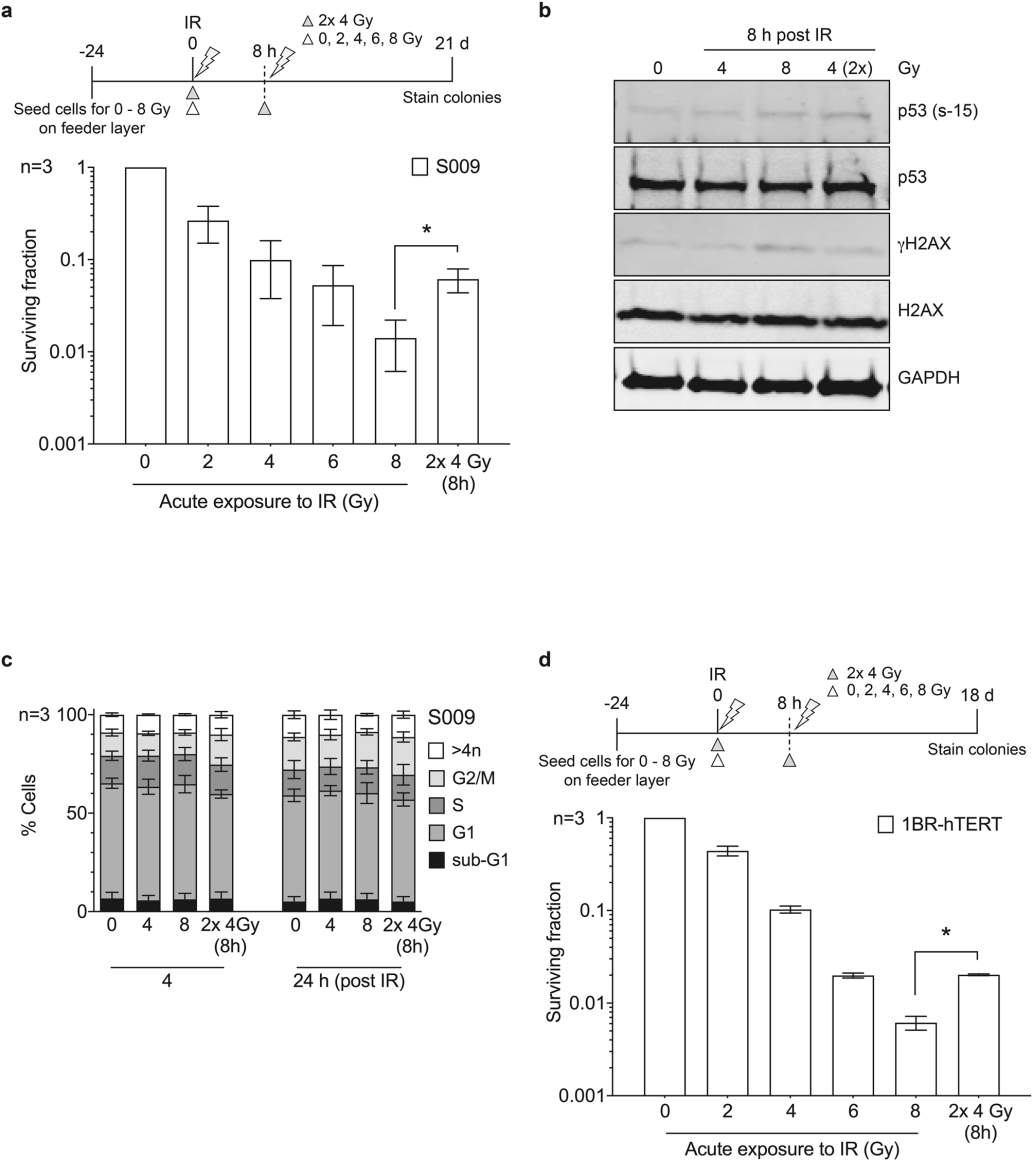
Primary breast fibroblast shows split-dose recovery. Breast fibroblast S009 cells were exposed to either acute or split-dose IR with indicated doses. (**a**) Colony survival assay shows significant split-dose recovery when 8 Gy is given as 2 doses of 4 Gy, 8 h apart (white triangle, single acute dose and grey triangle represents split-dose radiation) (**b**) Western blot analysis showing the expression levels of p53, H2AX and loading control GAPDH (full-length western blot images are presented in the Supplementary Fig. S9) (**c**) FACS analysis confirms majority of cells in G1 phase of the cell cycle 4 and 24 h after IR (4 and 24 h after the 2nd dose in split-dose experiments). (**d**) Colony survival assay of skin fibroblast 1BR hTERT confirms similar split-dose recovery.

We used normal primary human fibroblasts with intact DNA DSB machinery and WT p53 as representative of cells populating late-reacting normal tissues in patients. We confirmed that S009 primary breast skin fibroblasts show a significant split-dose recovery when the radiation dose is split into 2 smaller fractions of 4 Gy 8 h apart compared to a single acute dose of 8 Gy, with a RF of 5 ± 2.51 (Fig. 1a, Supplementary Fig. S1a, Table 1a). Radiation-induced expression levels of γH2AX remained higher 8 h after a single 8 Gy dose compared to when the dose was split into 2 smaller doses of 4 Gy, with expression levels similar to baseline after split-dose radiation, suggesting more efficient DNA repair (Fig. 1b). The S009 cells activated p53 after radiation exposure as shown by the formation of pS15 p53 demonstrating WT p53 function (Fig. 1b). Since p53 activates G1/S checkpoint arrest, we also examined cell cycle progression in S009 cells. We observed that cells remained predominantly in G1 phase of the cell cycle 4 and 24 h post–IR, consistent with an ability to activate p53 (Fig. 1c). As S009 cells are only capable of a limited number of passages, we aimed to reproduce this split-dose recovery in the well-characterised immortalised primary fibroblasts 1BR hTERT for further experiments, as confirmed in Fig. 1d (and Supplementary Fig. S1b). 1BR hTERT was previously shown to have a functional p53 and DNA damage response^25,26^.

**Table 1:** Summary of recovery factors (RF: ratio of split-dose to single dose survival) observed in selected primary and malignant cell lines (a) fibroblast and glioma cell lines, (b) ovarian and prostate cancer cell lines.

IR dose	Primary fibroblast cell lines				Glioma cell lines	
	S009 (WT p53)	1BR hTERT (WT p53 & DNA ligase IV)	MDAH041 (mut p53)	411BR hTERT (def DNA ligase IV)	M059K (WT DNA-PKcs)	M059J (def DNA-PKcs)
2 × 1 Gy vs 2 Gy	–	0.92 ± 0.13	–	1.21 ± 0.16	0.88 ± 0.08	0.55 ± 0.11
2 × 2 Gy vs 4 Gy	–	1.54 ± 0.35	–	1.16 ± 0.11	1.23 ± 0.26	0.38 ± 0.01
2 × 3 Gy vs 6 Gy	–	4.38 ± 1.31	–	1.33 ± 0.32	–	–
2 × 4 Gy vs 8 Gy	5.0 ± 2.51	3.5 ± 1.29	1.34 ± 0.28	–	–	–

	Ovarian cancer cell lines		Prostate cancer cell lines			
	A2780 WT (WT p53)	A2780 E6 (def p53)	IR dose	PC3 (mut p53)		
2 × 1 Gy vs 2 Gy	1.43 ± 0.16	1.16 ± 0.49	1.87 ± 0.19	0.77 ± 0.32		
3 × 1 Gy vs 3 Gy	2.95 ± 0.80	1.44 ± 0.66	1.91 ± 1.01	0.78 ± 0.13		
4 × 1 Gy vs 4 Gy	6.26 ± 3.54	1.82 ± 0.33	3.1 ± 1.30	1.20 ± 0.60		

All values are shown as mean ± standard deviation (n = 3).

### Fibroblasts with loss of functional p53 are fraction size insensitive, showing no split-dose recovery

**Figure 2:**
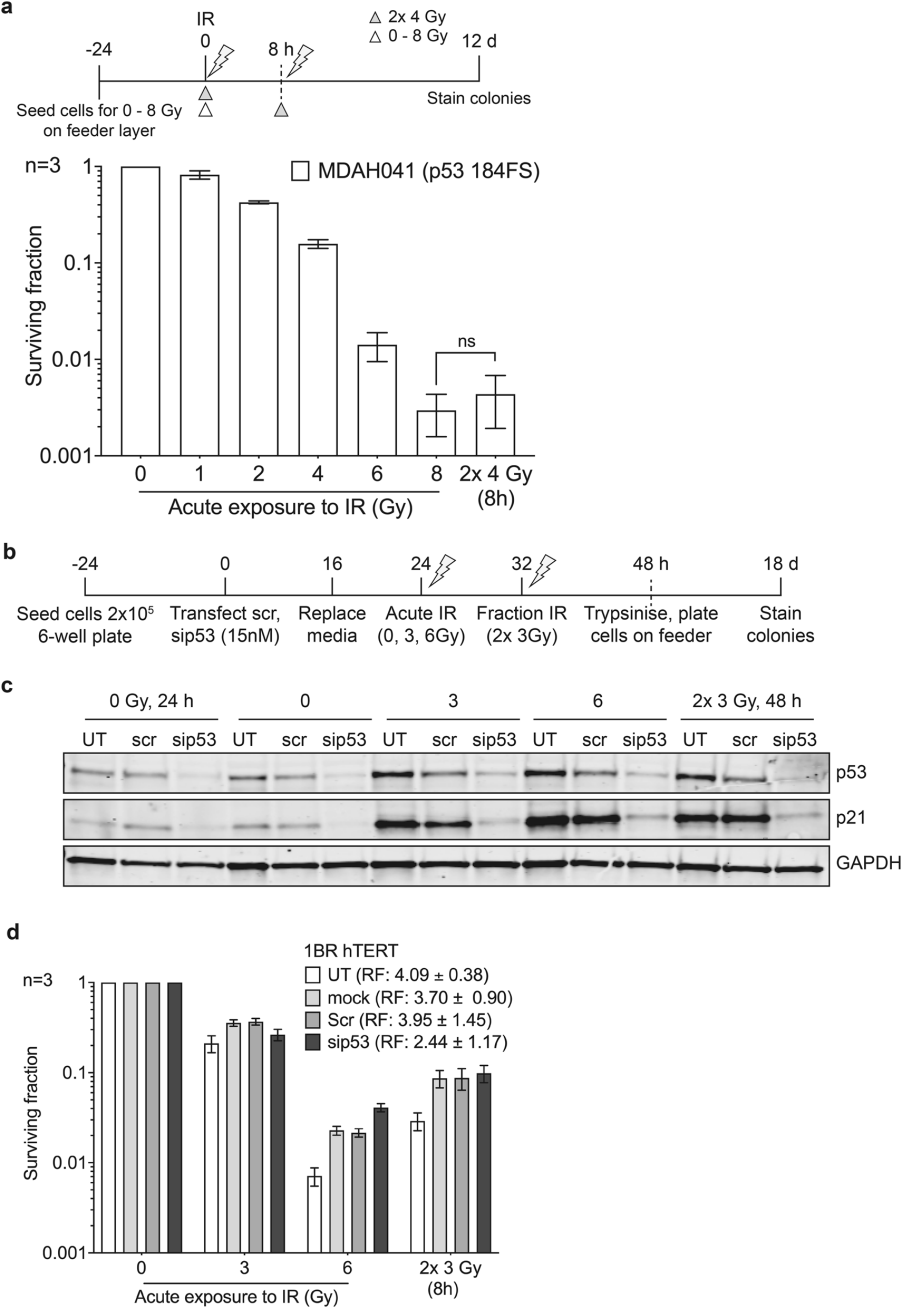
Split-dose recovery is not observed in primary fibroblast with loss of functional p53. Transformed Li-Fraumeni fibroblasts MDAH041 were exposed to either acute or split-dose IR with indicated doses (**a**) Colony survival assay confirms loss of split-dose recovery (white triangle, single acute dose and grey triangle represents split-dose radiation) (**b**) Schema for (**c**) western blot analysis showing expression levels of total p53, p21 and loading control GAPDH (full-length western blot images are presented in the Supplementary Fig. S10) and (**d**) colony survival of p53 siRNA knockdown in 1BR hTERT cells for the indicated period. UT is untreated, mock represents cells treated with DharmaFECT1 transfection reagent and Scr is the ON-TARGETplus non-targeting control scramble. RF, the ratio of split-dose to single dose survival, has been compared for each experimental condition. Western blot shown here is a representation of one of three individual experiments performed (Supplementary Fig. S4).

To test the effect of loss of functional p53 on split-dose recovery, we used transformed Li-Fraumeni cells (MDAH041). No significant split-dose recovery was observed in these cells (Fig. 2a, Supplementary Fig. S1c, Table 1a). Previous studies have demonstrated loss of p53 expression in MDAH041 cells^27,28^ and this was confirmed by western blot analysis with failure to increase p21 expression post-irradiation, indicative of loss in functional p53 (Supplementary Fig. S2). Post-irradiation, MDAH041 cells demonstrated a G2/M arrest in keeping with loss of G1/S checkpoint control (Supplementary Fig. S3).

To further test our hypothesis, we performed p53 knockdown by siRNA in 1BR hTERT cells (Fig. 2b). The knockdown of p53 expression was confirmed by western blotting (Fig. 2c & Supplementary Fig. S4). We observed that transient knockdown of p53 in 1BR hTERT cells led to reduced split-dose recovery with a RF of 2.44 ± 1.17 compared to 4.09 ± 0.38 in the untreated control following 2 smaller fractions of 3 Gy versus a single dose of 6 Gy, consistent with our hypothesis (Fig. 2d, Supplementary Fig. S1d).

### NHEJ-deficient primary fibroblasts lose split-dose recovery

**Figure 3:**
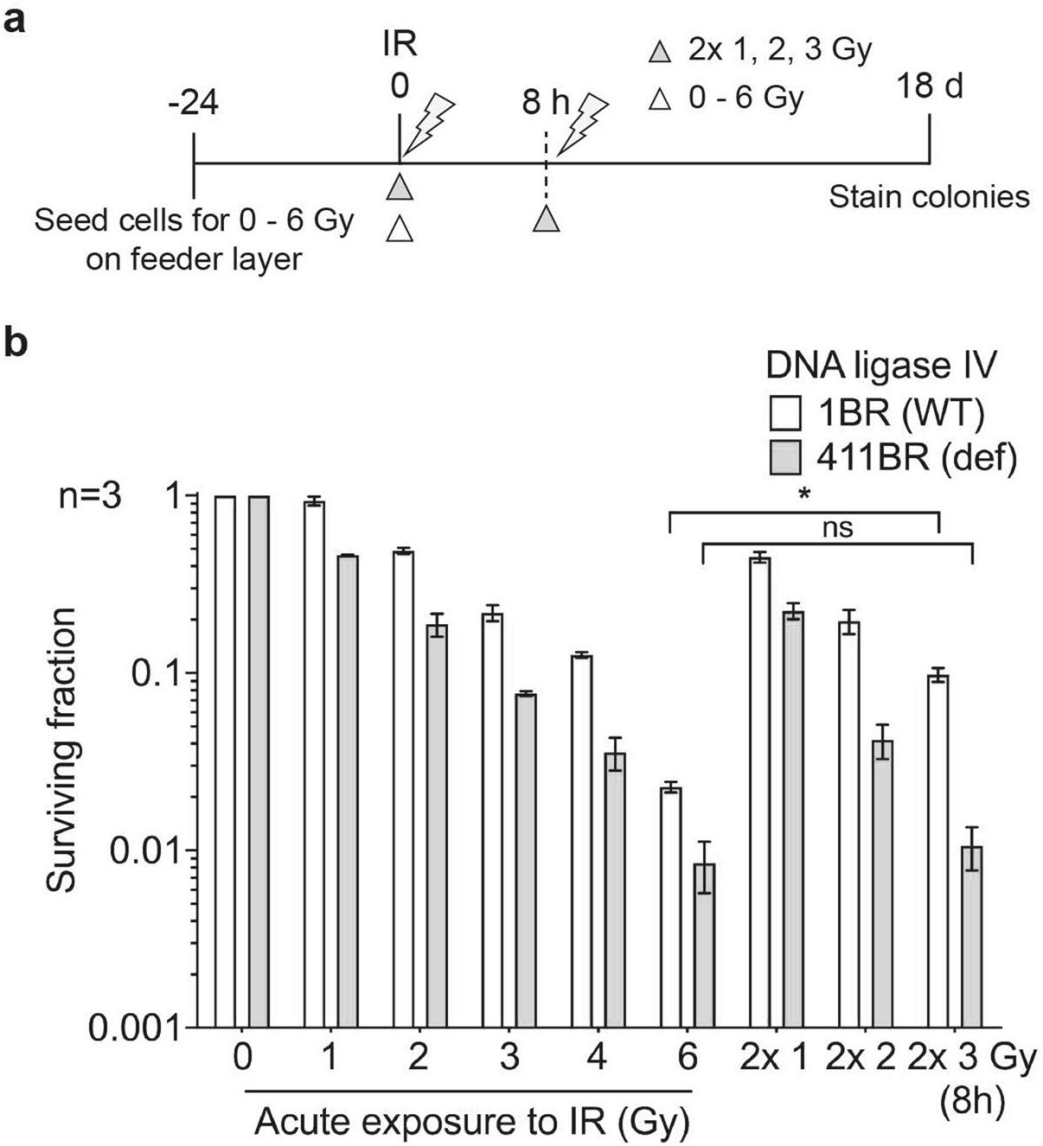
Reduced split-dose recovery is observed in NHEJ defective fibroblast cells. 1BR hTERT and 411BR hTERT fibroblast cells were exposed to either acute or split-dose IR with indicated doses (**a**) schema (white triangle, single acute dose and grey triangle represents split-dose radiation) (**b**) colony survival assay confirming loss of split-dose recovery in 411BR hTERT. ‘*’ indicating statistical significance between acute 6 Gy vs 2 × 3 Gy in 1BR hTERT cells.

We previously demonstrated that fraction size sensitivity is lost in NHEJ-deficient rodent cell lines^29^. In this study, we aimed to determine the influence of NHEJ repair on fraction size sensitivity in human primary fibroblasts using the NHEJ-deficient 411BR hTERT and its WT counterpart 1BR hTERT. At low acute doses of 2 and 4 Gy to the WT control, we were not able to see split-dose recovery using 2 × 1 Gy and 2 × 2 Gy, both probably being below the threshold to elicit a difference. However, when irradiated with 2 split doses of 3 Gy versus a single dose of 6 Gy, the 411BR hTERT cells showed loss of split-dose recovery compared to 1BR hTERT with RFs of 1.33 ± 0.32 and 4.38 ± 1.31, respectively (Fig. 3, Supplementary Fig. S1e, Table 1a).

### Tumour cells with loss of functional p53 are fraction size insensitive

**Figure 4:**
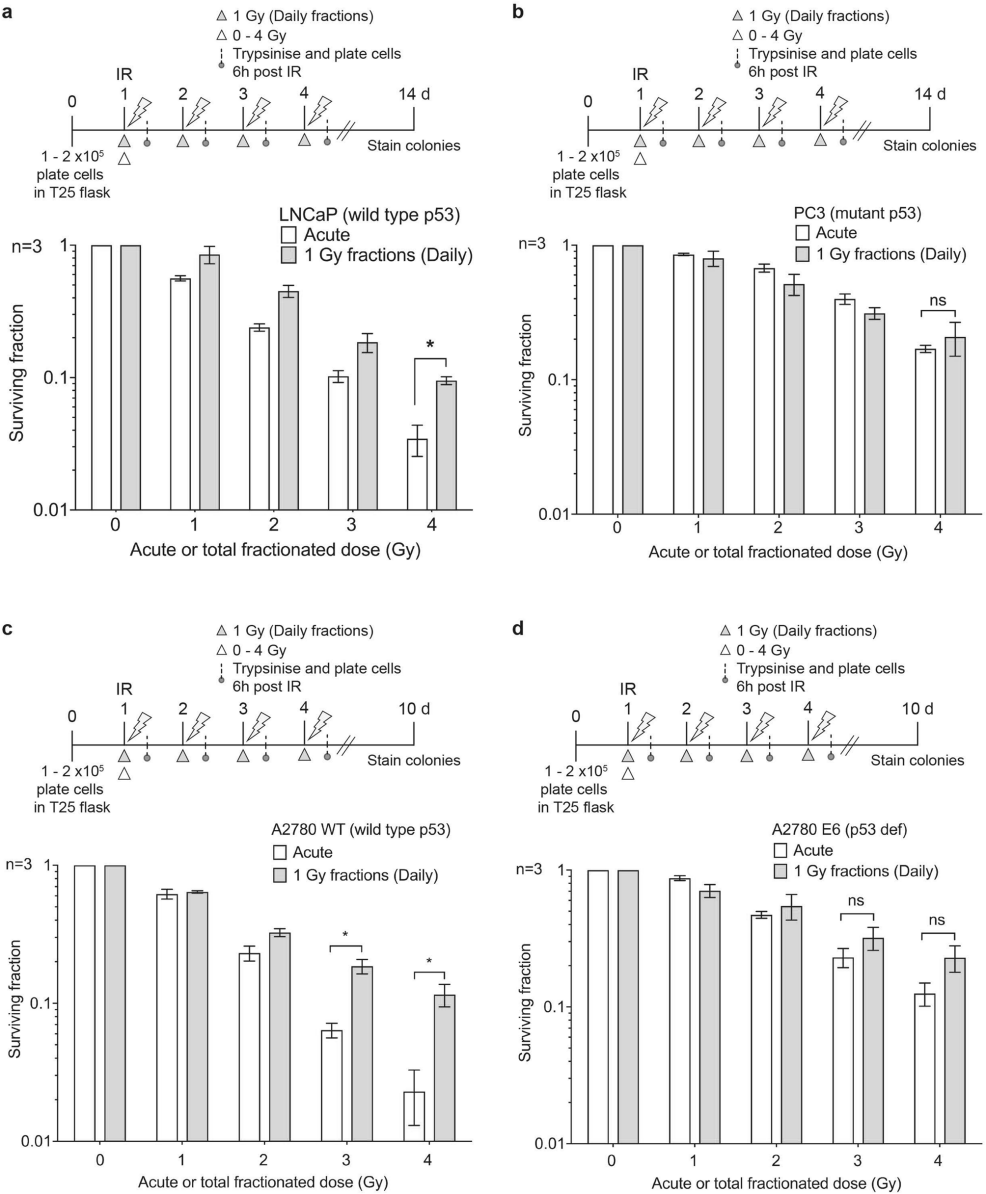
Split-dose recovery is lost in tumour cell lines with mutant p53. Colony survival of tumour cell lines (**a**) LNCaP (**b**) PC3 (**c**) A2780 WT and (**d**) A2780 E6 after exposure to either acute or daily fractionated IR with indicated doses. Top panel in each histogram shows the experimental schema, white triangle represents single acute dose and grey triangle 1 Gy daily fractions. Post radiation (6 h) cells were trypsinised and pooled with cells collected from media, plated and allowed to form colonies. Significant increase in split-dose recovery is observed in p53 WT tumour cell lines, LNCaP and A2780 WT (**a**,**c**) but not in mut p53 cell lines, PC3 and A2780 E6 (**b**,**d**).

Having demonstrated that p53 status influences fraction size sensitivity in normal cells, we went on to determine if the same was true for tumour cells. We tested prostate (LNCaP and PC3) and isogenic ovarian tumour cell lines (A2780 WT and A2780/E6 where p53 is silenced by HPV E6). Following fractionated irradiation, the p53 WT cells LNCaP and A2780 WT were fraction size sensitive with significant split-dose recovery (RF of 3.1 ± 1.30 and 6.26 ± 3.54, respectively). This was significantly reduced in the p53-defective PC3 and A2780/E6 cells (RF of 1.20 ± 0.60 and 1.82 ± 0.33, respectively) (Fig. 4, Supplementary Fig. S1f–g & S5, Table 1b). There was a significant difference in the cell cycle profiles of these cells with p53 WT cells (LNCaP and A2780 WT) mainly in G1 phase of the cell cycle in contrast to the p53-deficient cells (PC3 and A2780/E6) that were predominantly in S/G2 phase post-radiation (Supplementary Figs. S6, S7). This is consistent with published literature assessing cell cycle distribution following multi-fraction radiation in LNCaP and PC3^30^.

### NHEJ defective glioma cells are fraction size insensitive

**Figure 5:**
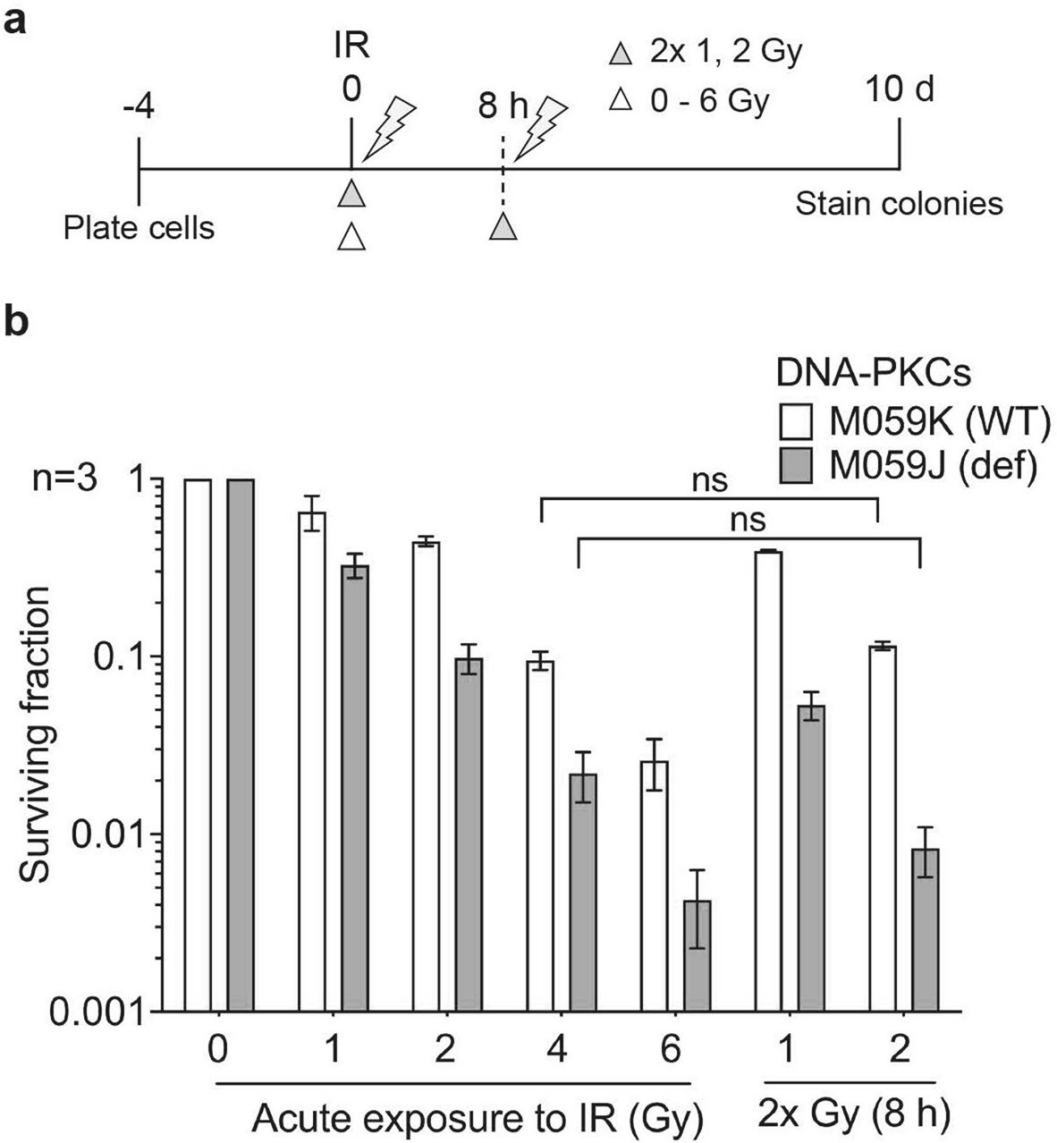
Split-dose recovery is reduced in DNA-PKcs defective glioma cells. Glioma cells M059K and M059J were exposed to either acute or split-dose IR with indicated doses (**a**) Schema (white triangle, single acute dose and grey triangle represents split-dose radiation) (**b**) colony survival assay shows increased radiosensitivity and reduced split-dose recovery.

To study the effects of NHEJ deficiency in a tumour model, we used the p53-mutant glioma cell lines proficient (M059K) and defective (M059J) for DNA-PKcs. We hypothesised that the DNA-PKcs defective cell line would be fraction size insensitive compared to its WT counterpart. However, neither of these cell lines showed significant split-dose recovery (Fig. 5, Supplementary Fig. S1h, Table 1a). In fact, M059J showed increased sensitivity to split doses 8 h apart, suggesting that unrepaired DSBs from the first dose sensitised these cells to the second dose. Treating M059K cells with a DNA-PKcs inhibitor reproduced the findings in M059J cells with increased radiosensitivity and no evidence of split-dose recovery (Supplementary Fig. S8). Notably, both cell lines have a p53 mutation^31^. The lack of difference observed in the WT and DNA-PKcs defective cells is likely to be the result of loss of p53 function in both cell lines. The results suggest that tumour cells with p53 mutation, regardless of whether or not they have intact NHEJ, are likely to be fraction size insensitive.

## Discussion

We show for the first time in a range of normal and malignant human cell lines that the sparing effect of small fractions on cell survival is dependent on functional p53 and that sparing is lost/reduced if p53 is mutated or transiently knocked down. To clarify the role of p53 and NHEJ, in response to fractionated radiation we established split-dose recovery by exposing the same cell line to both single and fractionated radiation whilst maintaining an identical genetic and repair machinery in the background. Our findings are consistent with a study testing tumour growth delay in two genetic variants of a lung adenocarcinoma mouse model after either a single fraction of 11.6 Gy or two fractions of 7.3 Gy in which no significant difference in the response of tumours deficient in p53 (LSL-Kras; p53^FL/FL^ mice) were seen after single or two smaller fractions (p = 0.23), in contrast to tumours with WT p53 (p = 0.002)^32^. In contrast, Scott et al. reported that following 2 Gy daily fractions over 5 days, the prostate cell line LNCaP showed reduced survival compared to PC3^30^. However, a significant drawback in this study is that the authors failed to compare fractionated dose to the corresponding single acute dose survival, instead their results were based on predicted survival. Similarly, Eke et al. reported conflicting results, with LNCaP showing less sparing with fractionated doses compared to PC3. Interestingly, they demonstrated that mouse embryonic fibroblasts with WT p53 had increased survival following fractionated radiation and this was mediated by 53BP1, a cellular factor that binds p53^33^. Cells with a higher recovery factor were shown to have lower residual gH2AX and 53BP1 foci consistent with DNA repair having a role in determining fraction size sensitivity^33^.

TP53 is one of the most commonly mutated genes in cancer and known to have functional role in the modulation of G1/S, G2/M checkpoint, DNA repair, cell death and senescence^34^. The frequency of somatic TP53 mutation varies significantly across solid tumours^35^. The Cancer Genome Atlas (TCGA) reports somatic TP53 mutations in up to 81% of lung and 72% of head and neck squamous cell carcinomas, at one extreme, with a decreasing frequency of 46% in lung adenocarcinoma, 37% in breast carcinoma and 8% in prostate cancer^36–41^. It is striking that this distribution of p53 mutation frequency mirrors the known average fraction size sensitivities of these tumour types (Supplementary Table S1). Breast and prostate cancers have the lowest rates of p53 mutations and the highest levels of sensitivity to fraction size.

NHEJ is error prone and fidelity of repair is sensitive to fraction size^42^. In contrast, HR is known to have high fidelity and there is no evidence that this fidelity is influenced by dose. A linear-quadratic increase with fraction size of lethal unbalanced chromosome exchanges has already been referred to as underlying the sensitivity of cells to fraction size in G0/1 human fibroblasts, in which NHEJ is the dominant determinant of DSB repair/misrepair^15,16^. If sensitivity to fraction size is a function of the ability to undertake NHEJ repair, the challenge is to understand how this sensitivity is weakened. It has long been known that tumour cell lines defective in p53 tend to have higher proliferation rates compared to those with WT p53^43–46^. In the cells we tested, PC3 having defective p53 has a doubling time of 33 h compared to the slow growing LNCaP with WT p53 and a doubling time of 60 h^47,48^. The A2780 WT p53 cells have a doubling time of 18–22 h, compared to much shorter doubling times for A2780/E6 with mut p53^49,50^.

Here, we also demonstrated that 411BR hTERT human fibroblasts deficient in DNA Ligase IV, a key component of NHEJ, show loss of fraction size sensitivity with no spilt-dose recovery compared to the WT 1BR3 hTERT fibroblasts. This is consistent with a recent study which has shown that 53BP1-mediated NHEJ has a clear role in increased survival of both cancer and mouse embryonic fibroblast cells post-fractionated radiation^33^. Our results in the glioma cell lines proficient (M059K) and defective (M059J) for DNA-PKcs suggest that tumour cells with p53 mutation, regardless of whether or not they have intact NHEJ, are likely to be fraction size insensitive. These observations are consistent with loss of fractionation sensitivity in p53-mutant CHO cell lines defective in NHEJ which rely on HR repair with cells accumulating in S/G2 phase of the cell cycle with fractionated RT^29,51,52^.

In normal tissues, there is a tight association between proliferative indices and fraction size sensitivity^18^. The association is so close as to suggest mechanistic links. Early human skin reactions (erythema and desquamation) depend strongly on total dose, but they are less sensitive to fraction size than the late onset side-effects that are commonly dose-limiting^53^. We, and others, have shown that over a 5-week course of radiotherapy basal epidermal cells accumulate in S/G2 phase and show a tenfold increase in RAD51 foci compared to baseline, consistent with the idea that high fidelity HR repair of radiation-induced DNA DSBs contributes to the weak sensitivity to fraction size of human epidermis^54–56^. On this basis, the loss of recovery in p53mut tumour cells may be a function of greater reliance on high fidelity HR.

Although randomised clinical trials confirm wide differences in average fraction sensitivity between adenocarcinomas of the breast and prostate on one hand and squamous carcinomas of the lung and head and neck on the other, they provide no measure of variation in fraction sensitivity between tumours arising at the same anatomical site. A potentially important therapeutic outcome of the findings in our study is that tumours retaining an intact p53 pathway and, thereby, a proficient G1/S checkpoint are as sensitive to fraction size as dose-limiting normal tissues, undermining the clinical rationale for small fractions in such tumours. If these pre-clinical observations hold true in human tumours, it may be possible to improve radiotherapy response by using hypofractionated schedules in p53 WT tumours and conventional fractionation to a higher total dose in p53-mutant tumours.

In conclusion, our data support the hypothesis that cells defective in p53 are less sensitive to RT fraction size. The loss of split-dose recovery observed in DNA ligase IV deficient cells is interpreted as the dependence of fraction size sensitivity on intact NHEJ. This offers an explanation for loss of fraction size sensitivity in rapidly dividing malignant and normal cells with important implications for personalized radiotherapy dose prescriptions and biomarker development.

## Supplementary Information

Supplementary Information.

## Data Availability

Data from this study are available from the corresponding author upon request.
