# Supplementary material for: TP53 modulates radiotherapy fraction size sensitivity in normal and malignant cells
Source: Sci Rep. 2021 Mar 29;11:7119. doi: 10.1038/s41598-021-86681-6 (PMC8007815; doi:10.1038/s41598-021-86681-6)
Supplement: Supplementary file 1 — Supplementary Information. [file 41598_2021_86681_MOESM1_ESM.pdf]

TP53 MODULATES RADIOTHERAPY FRACTION SIZE SENSITIVITY IN NORMAL AND MALIGNANT CELLS

Selvakumar Anbalagan PhD[1], Cecilia Ström PhD[1], Jessica A Downs PhD[1], Penny A Jeggo PhD[1,3], David McBay BSc[1], Anna Wilkins FRCR[1,2], Kai Rothkamm PhD[4], Kevin J Harrington PhD[1,2], John R Yarnold FRCR[1,2] and Navita Somaiah DPhil[1,2]

[1]The Institute of Cancer Research, London, UK
[2]The Royal Marsden NHS Foundation Trust, London, UK
[3]Genome Damage and Stability Centre, University of Sussex, UK
[4]University Medical Centre Hamburg-Eppendorf, Germany

Corresponding author:
Dr Navita Somaiah, Team leader Translational Breast Radiobiology and Consultant Clinical Oncologist,
The Royal Marsden, Downs Road, Sutton SM2 5PT, UK
Tel: +44 20 89156501, Email: navita.somaiah@icr.ac.uk

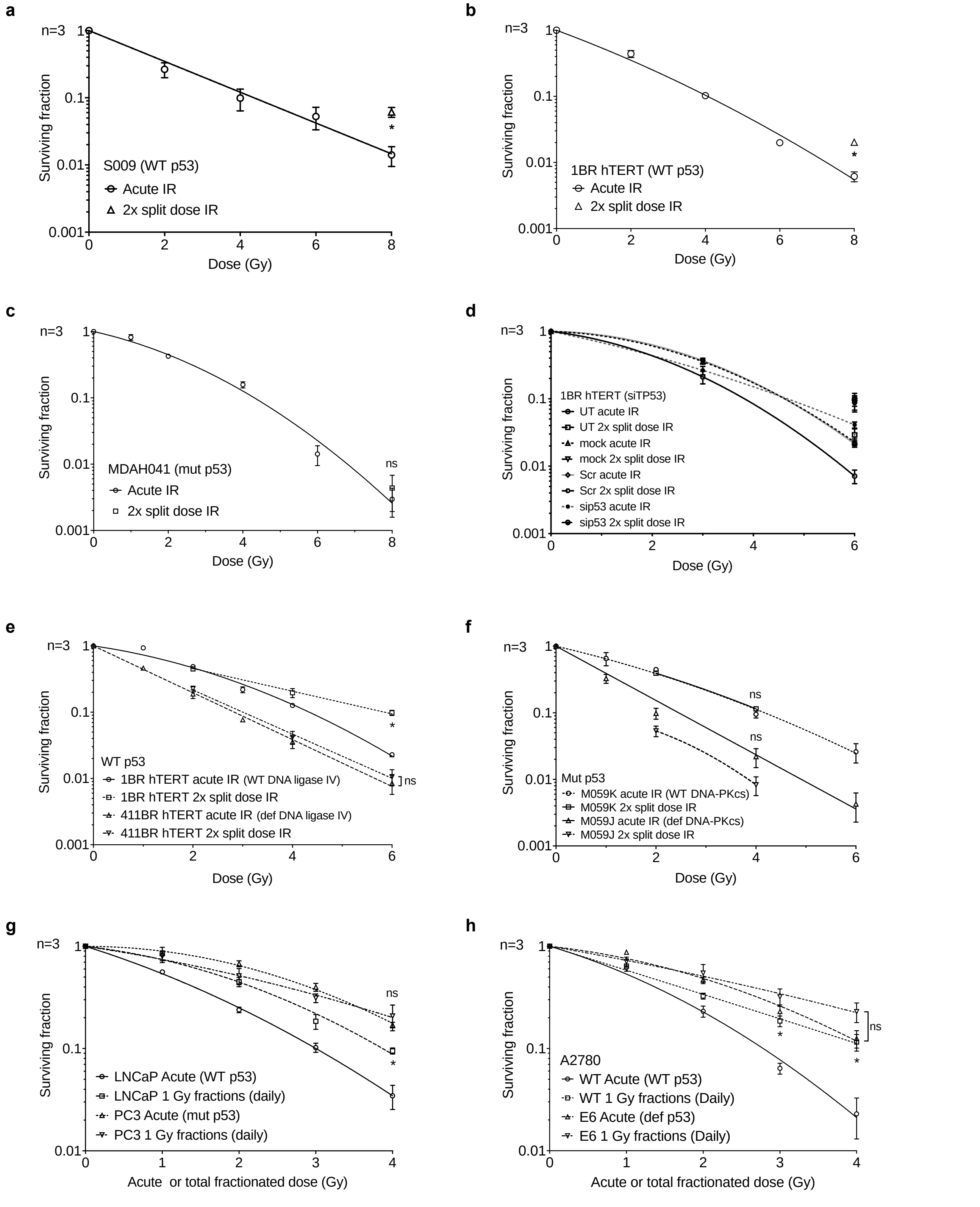

Fig S1. Colony survival of indicated cell lines w.r.t. their corresponding histograms in the manuscript fitted according to the equation SF=exp(-αD+βD^2) using Graphpad prism 7.0d.

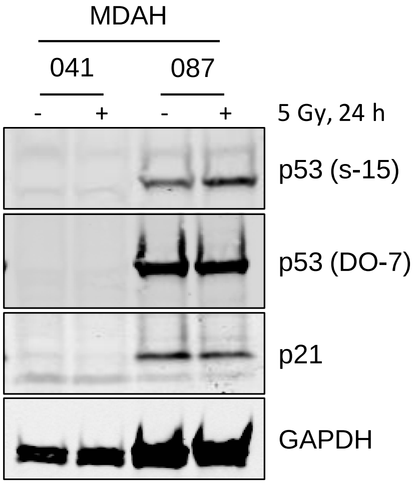

Fig S2. Western blot analysis of Li Fraumeni cell lines
Western blot analysis of MDAH041 (p53 184FS) and MDAH087 (p53 R24W) Li fraumeni cell lines (kind gift from Prof. Tainsky, Karamanos Cancer Institute) showing the expression levels of p53, p21 and loading control GAPDH, 24h post 5 Gy IR. MDAH087 was used as a positive control in view of GOF mutation[#]. Full-length western blot images are presented in the Supplementary Fig. S11.

[#]Zhu *et al*., (2015) Nature 525, 206-211.

a

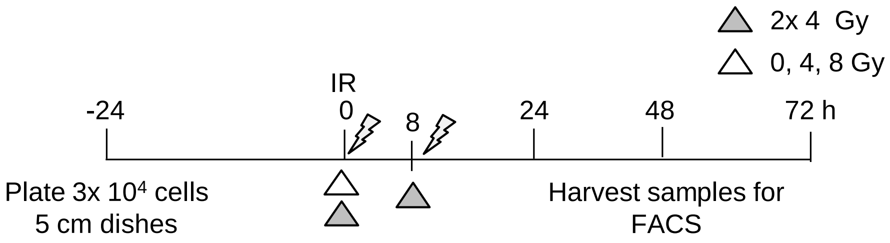

b

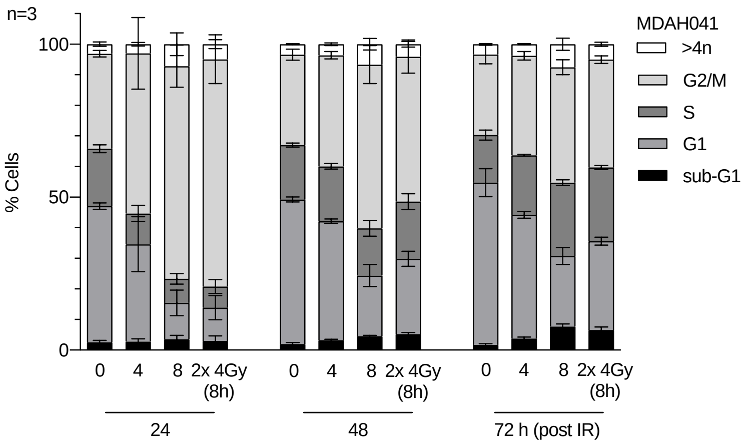

Fig S3. Cell cycle distribution in Li fraumeni cells post fractionated irradiation
(**a**) Schema of FACS analysis of (**b**) MDAH041 showing the percent cells in various cell cycle phases when exposed to either acute (white triangle) or fractionated irradiation (4 Gy fractions; 8 h apart; grey triangle) for the indicated doses and period.

a

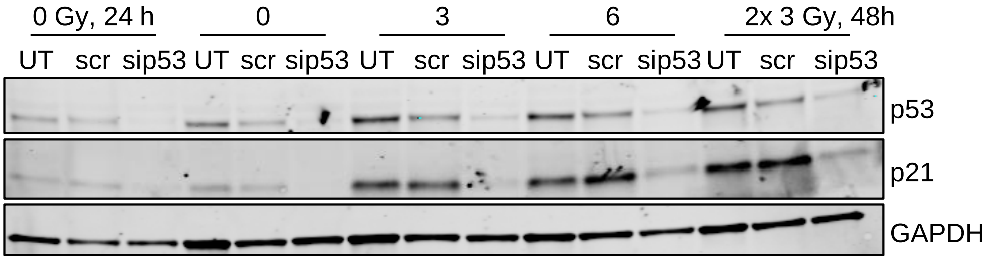

b

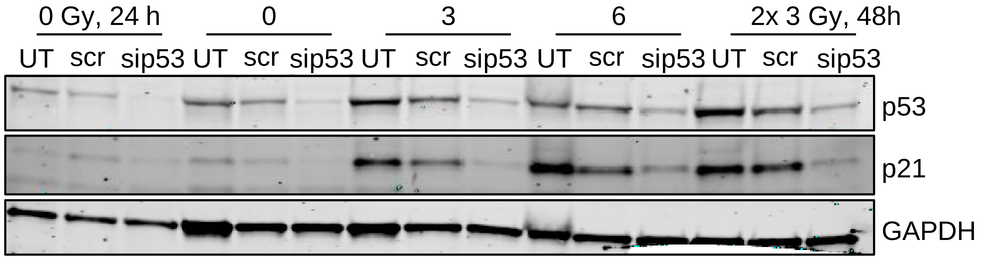

Fig S4. Western blot analysis showing the expression level of p53 knockdown in 1BR hTERT Western blot showing the expression levels of p53, p21 and the loading control GAPDH following p53 siRNA knockdown for the indicated period and IR doses performed in experiment 1(i) and repeat experiments 2(**S4a**) and 3 (**S4b**). 'scr' is ON-TARGETplus non-targeting control scramble as indicated in the materials and methods. Full-length western blot images are presented in the Supplementary Fig. S10.

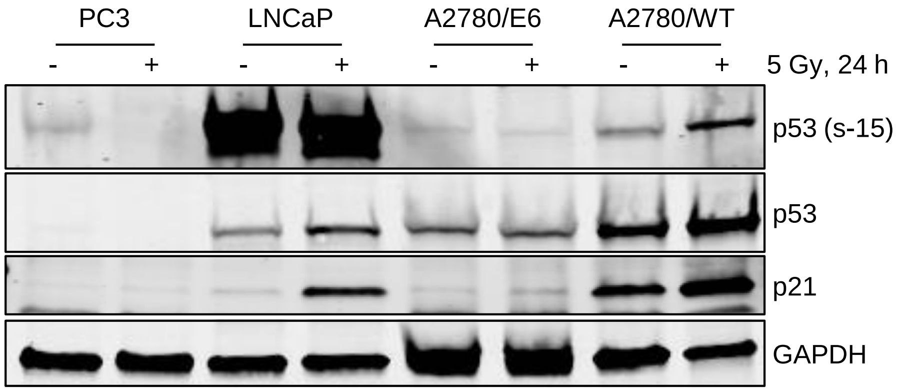

Fig S5. Western blot analysis of prostate and ovarian cancer cell lines post irradiation Western blot analysis of a panel of indicated cancer cell lines showing the expression levels of p53, p21 and loading control GAPDH, 24h post 5 Gy IR. Full-length western blot images are presented in the Supplementary Fig. S12.

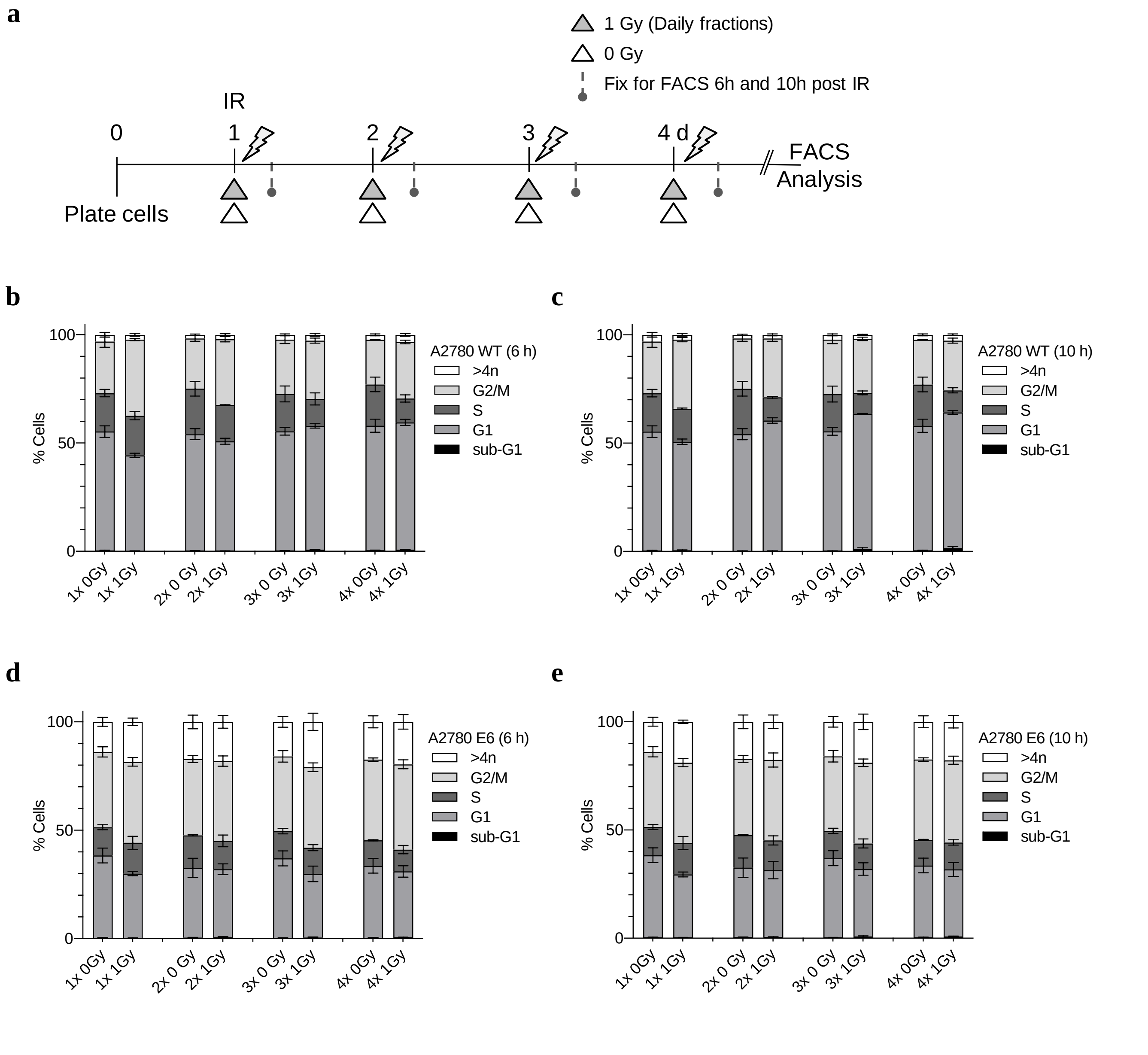

Fig S6. Cell cycle distribution in ovarian cell lines post irradiation

(**a**) Schema of FACS analysis showing daily 1 Gy fractions (grey triangle) and 0 Gy (white triangle) control cells. A2780 WT (**b** & **c**) and A2780/E6 cells (**d** & **e**) showing the percent cells in various cell cycle phases 6 and 10 h after exposure to daily fractions of 1 Gy for the indicated period (a).

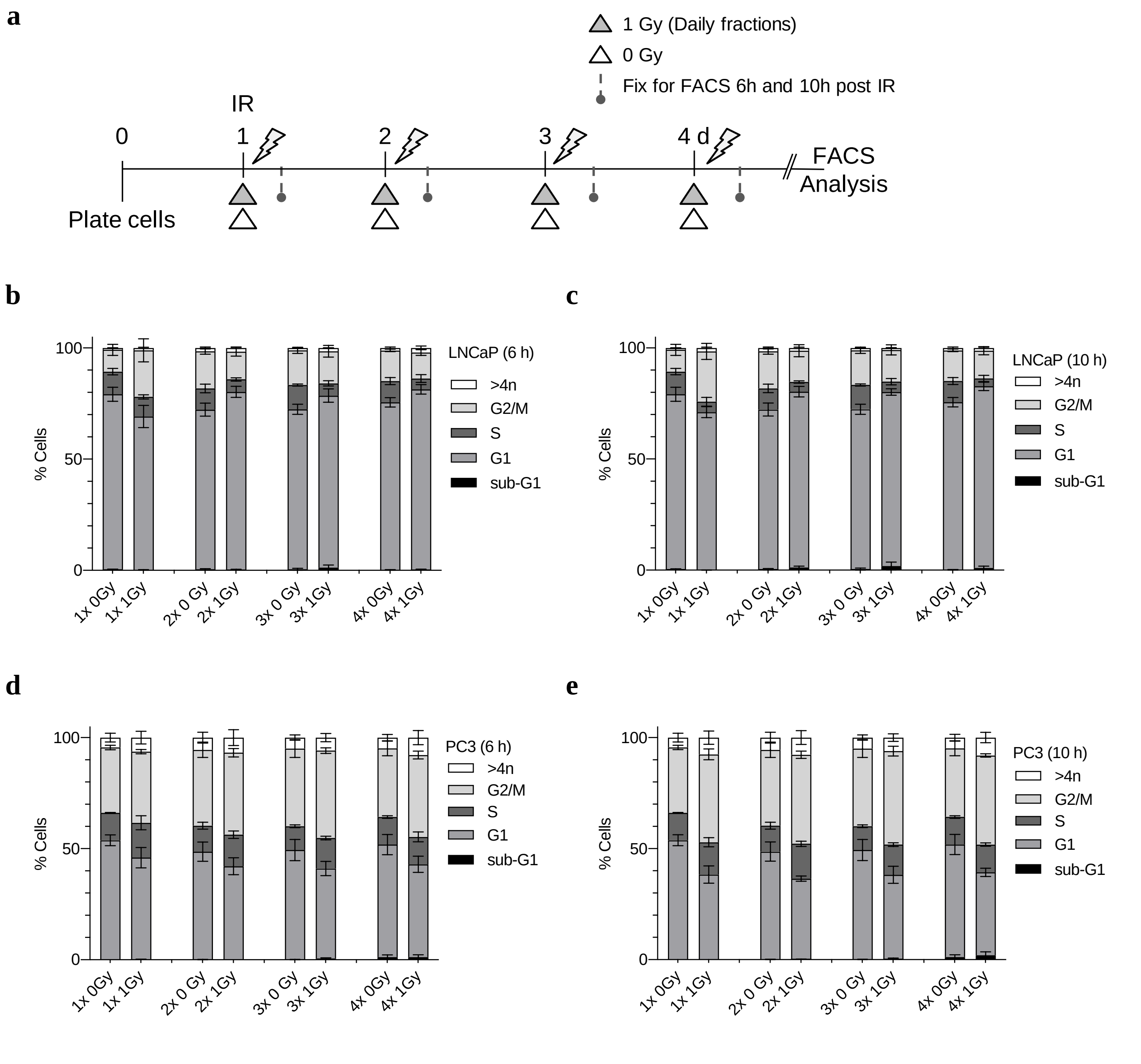

Fig S7. Cell cycle distribution in prostate cancer cell lines post irradiation

(**a**) Schema of FACS analysis showing daily 1 Gy fractions (grey triangle) and 0 Gy (white triangle) control cells. LNCaP (**b & c**) and PC3 (**d & e**) showing the percent cells in various cell cycle phases 6 and 10 h after exposure to daily fractions of 1 Gy for the indicated period (a).

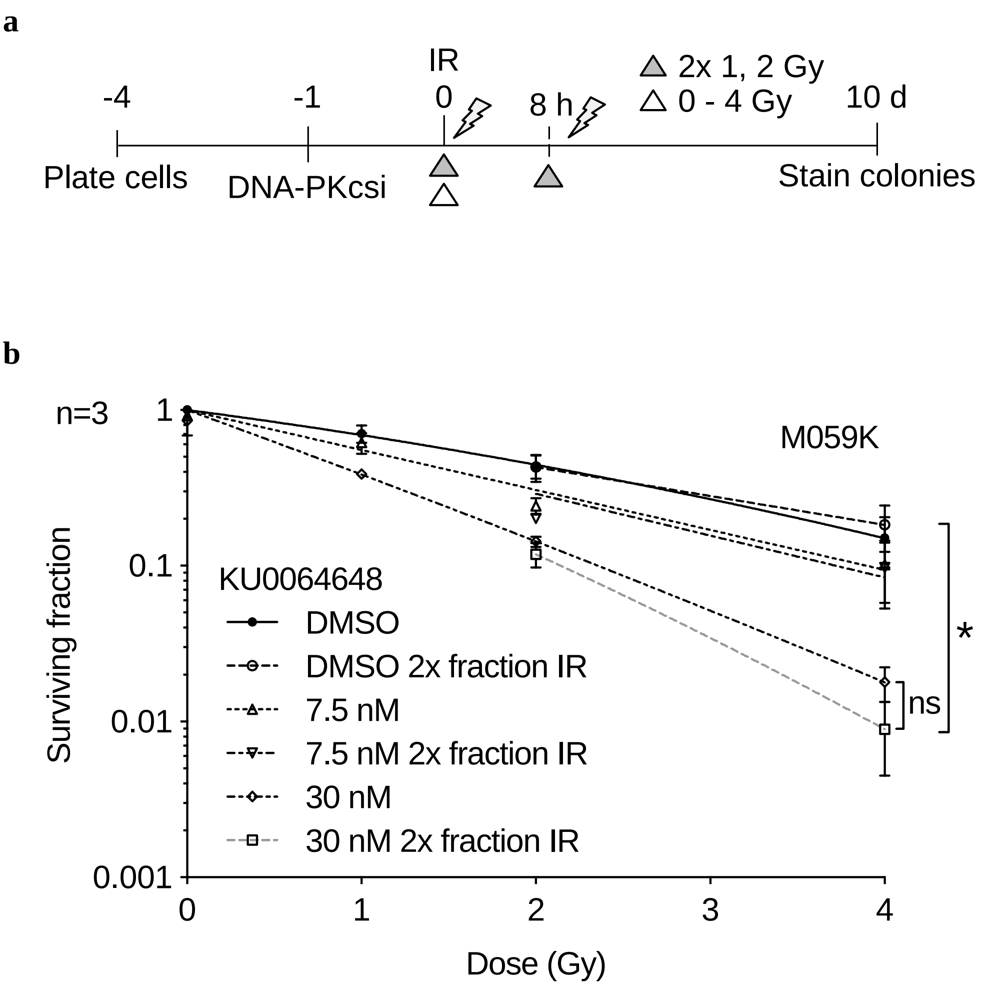

Fig S8. Treatment with DNA-PKcs inhibitor KU0064648 in M059K glioma tumour cell lines. (**a**) Schema of split dose colony survival assay in (**b**) M059K cells treated in combination with either DMSO or indicated doses of KU0064648 and exposed to either acute (white triangle) or fractionated IR doses (1 or 2 Gy fractions; 8 h apart; grey triangle) as indicated.

Table S1 List of somatic TP53 mutation in different tumour types (Cancer genome atlas)

Tumour types	Somatic TP53 mutation
Lung squamous cell carcinoma[1]	81%
HNSC[2]	72%
Lung adenocarcinoma[3]	46%
Breast carcinoma[4]	37%
Prostate[5]	8%

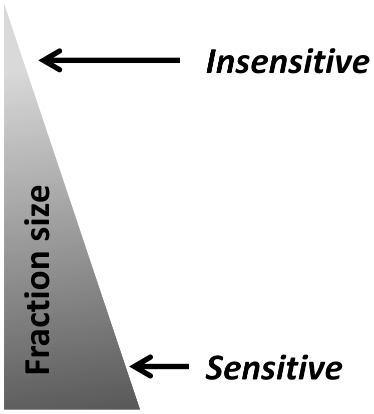

[1]Cancer Genome Atlas Research Network (2012), Nature
[2]Cancer Genome Atlas Research Network (2015), Nature
[3]Cancer Genome Atlas Research Network (2014), Nature
[4]Cancer Genome Atlas Research Network (2012), Nature
[5]Cancer Genome Atlas Research Network (2015), Cell

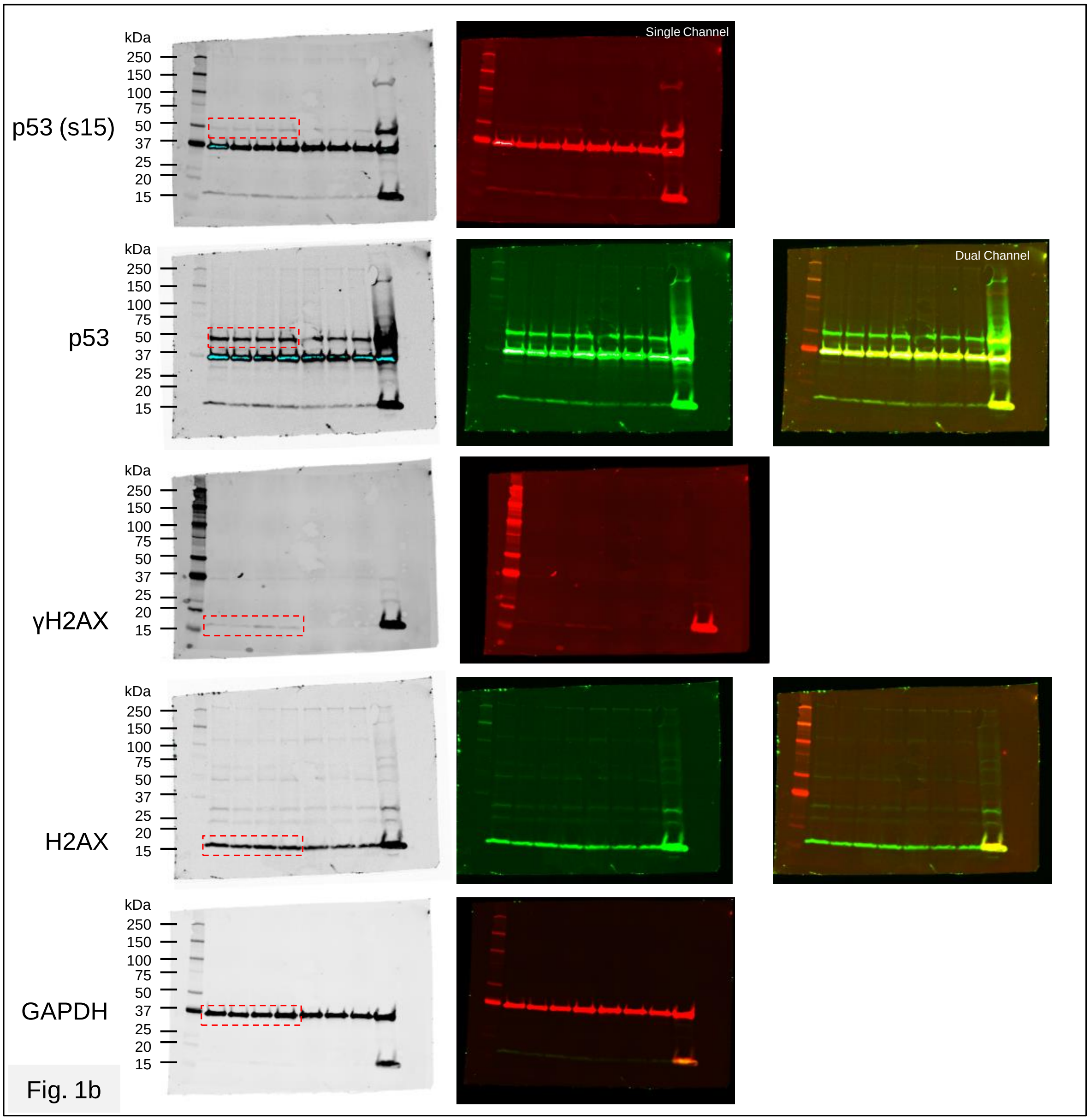

Fig S9. Uncropped western blot membrane whole scans related to the Fig. 1b presented in this study. Dotted red box represents the corresponding protein of interest for indicated conditions as shown in their respective western blot lane in Fig. 1b.

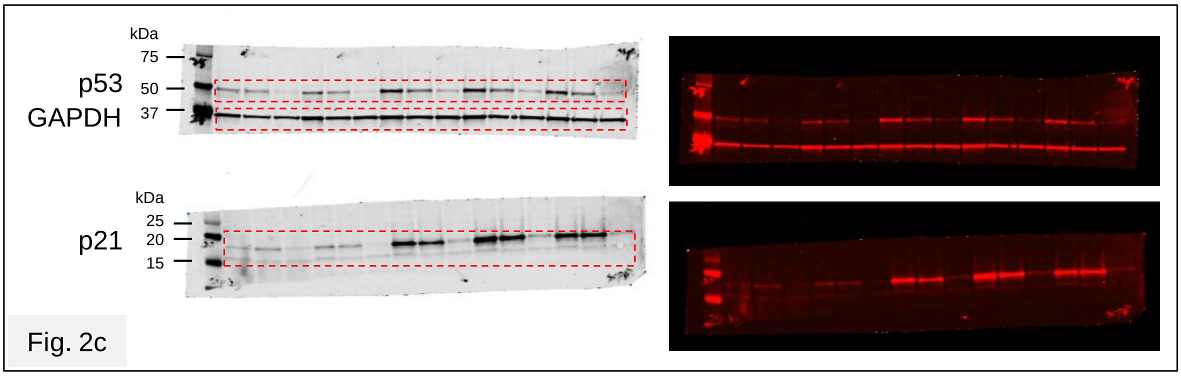

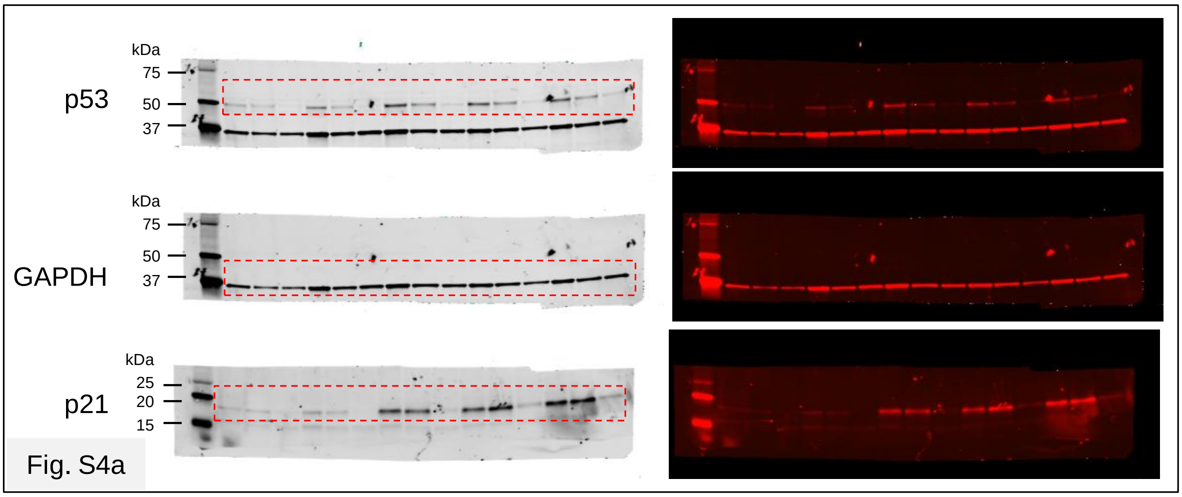

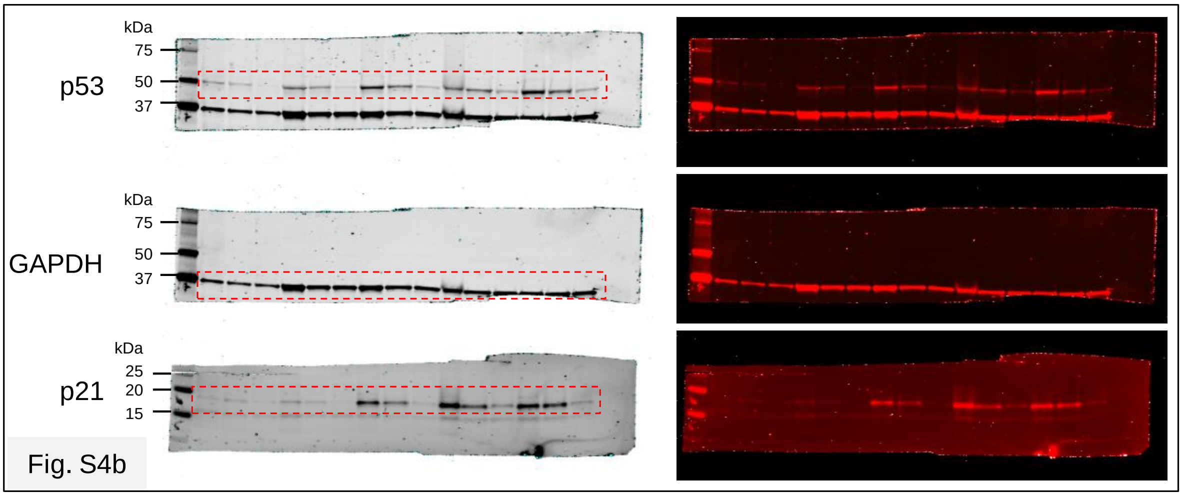

Fig S10. Uncropped western blot membrane whole scans related to Fig. 2c, S4a-b presented in this study. Dotted red box represents the corresponding protein of interest for indicated conditions as shown in their respective western blot lane in Fig. 2c, S4a-b.

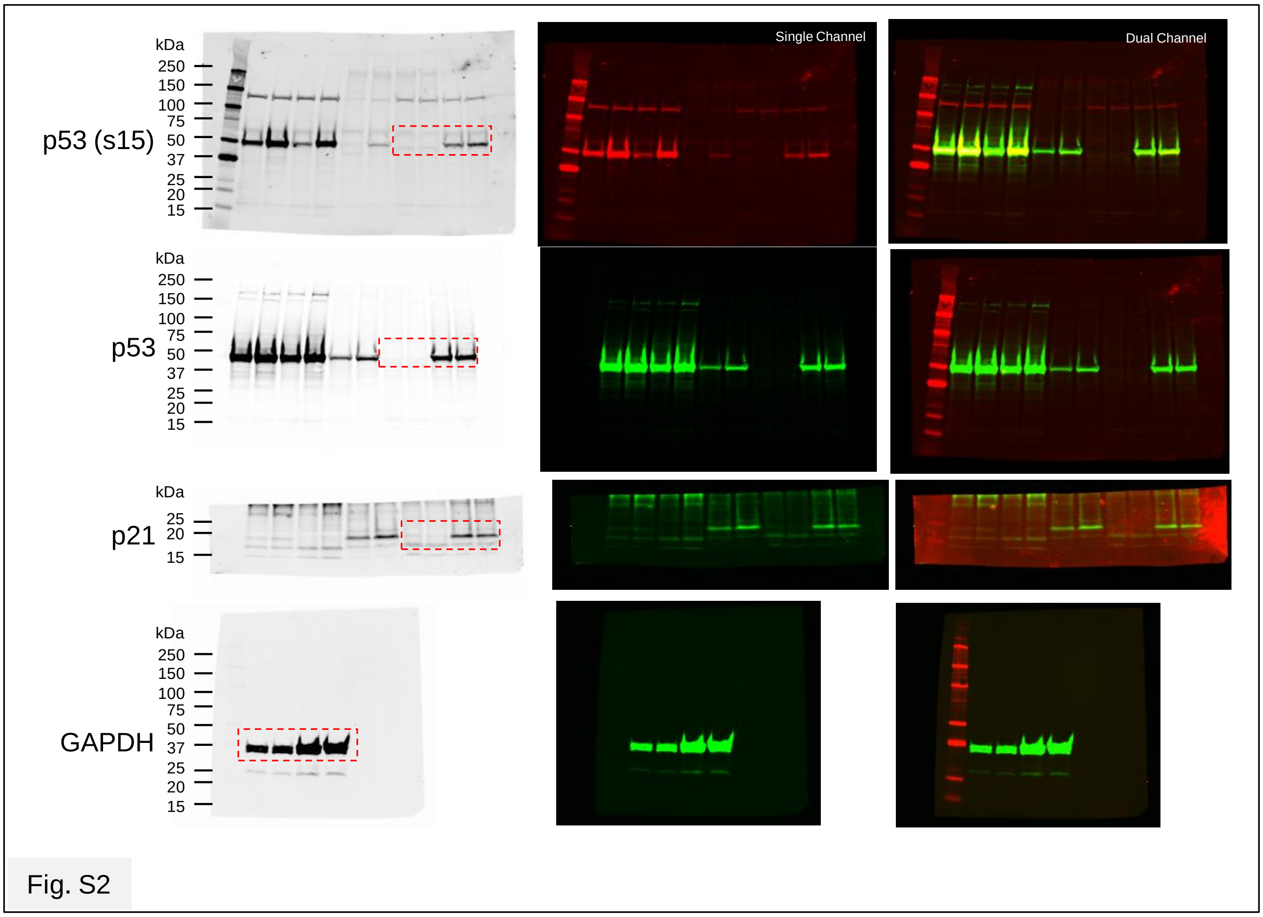

Fig S11. Uncropped western blot membrane whole scans related to Supplementary Fig. S2 presented in this study. Dotted red box represents the corresponding protein of interest for indicated conditions as shown in their respective western blot lane in Supplementary Fig. S2.

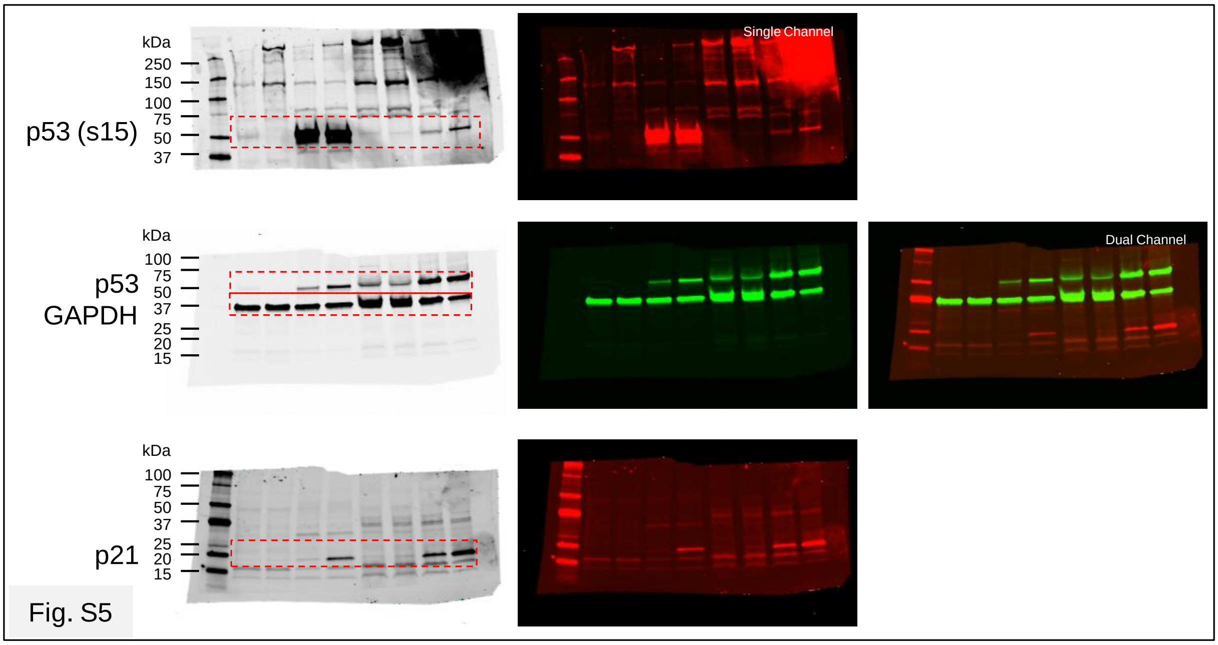

Fig S12. Uncropped western blot membrane whole scans related to Supplementary Fig. S5 presented in this study. Dotted red box represents the corresponding protein of interest for indicated conditions as shown in their respective western blot lane in Supplementary Fig. S5.

Table showing the list of antibodies along with their dilutions used in this study

Antibody	Species	Catalogue	Dilution	Distributor
Phospho-H2A.X (ser139), clone JBW301	Mouse	05-636	1:5000	Millipore
Anti-Histone H2A.X	Rabbit	AB10022	1:10000	Millipore
p21	Rabbit	2947	1:1000	Cell Signalling
GAPDH (6C5)	Mouse	NB600-502	1:5000	Novus Bio
Phospho-p53 (s15)	Rabbit	9284	1:1000	Cell Signaling
p53 DO-7	Mouse	M7001	1:1000	Dako
IRDye 800CW Donkey Anti-Mouse IgG (H+L), 0.5 mg	Mouse	926-32212	1:5000	Li-Cor
IRDye 800CW Donkey Anti-Rabbit IgG (H+L), 0.5 mg	Rabbit	926-32213	1:5000	Li-Cor
Alexa Fluor 680 goat anti-mouse IgG (H+L) *2 mg/mL	Mouse	A21057	1:10000	Invitrogen
Alexa Fluor 680 goat anti-rabbit IgG (H+L) *2 mg/mL	Rabbit	A21076	1:10000	Invitrogen
